# 21st century headache: mapping new territory

**DOI:** 10.1186/s10194-021-01233-7

**Published:** 2021-04-01

**Authors:** Peter J. Goadsby, Michel Lantéri-Minet, Martin C. Michel, Mario Peres, Mamoru Shibata, Andreas Straube, Tissa Wijeratne, Caty Ebel-Bitoun, Luminita Constantin, Simon Hitier

**Affiliations:** 1 NIHR-Wellcome Trust King’s Clinical Research Facility, King’s College Hospital, London, SE5 9PJ UK; 2 Department of Neurology, University of California, Los Angeles, USA; 3 Pain Department and FHU InovPain, CHU Nice – Côte Azur Université, Nice, France; 4 INSERM U1107 Migraine and Trigeminal Pain, Auvergne University, Clermont-Ferrand, France; 5 Department of Pharmacology, Johannes Gutenberg University, Mainz, Germany; 6 Universidade Federal de São Paulo, São Paulo, Brazil; 7 Department of Neurology, Tokyo Dental College Ichikawa General Hospital, Chiba, Japan; 8 Department of Neurology, Ludwig-Maximilians University, Munich, Germany; 9 AIMSS, Department of Neurology, Melbourne Medical School, Sunshine Hospital, Western Health, The University of Melbourne, Melbourne, Australia; 10 Sanofi, 82, Avenue Raspail, 94255 Gentilly Cedex, France

**Keywords:** 21st century headache, Triggers, Cognitive functioning, Over-the-counter medication, Real world evidence, Infodemiology

## Abstract

**Background:**

With headache experienced by up to 75% of adults worldwide in the last year, primary headache disorders constitute a major public health problem, yet they remain under-diagnosed and under-treated.

Headache prevalence and burden is changing as society evolves, with headache now occurring earlier in life. Contributing factors, mostly associated with changing life style, such as stress, bad posture, physical inactivity, sleep disturbance, poor diet and excess use of digital technology may be associated with the phenomenon that could be labelled as ‘21st century headache’. This is especially notable in workplace and learning environments where headache impacts mental clarity and therefore cognitive performance. The headache-related impact on productivity and absenteeism negatively influences an individual’s behaviour and quality of life, and is also associated with a high economic cost. Since the majority of sufferers opt to self-treat rather than seek medical advice, substantial knowledge on headache prevalence, causation and burden is unknown globally. Mapping the entire population of headache sufferers can close this knowledge gap, leading to better headache management. The broad use of digital technology to gather real world data on headache triggers, burden and management strategies, in self-treated population will allow these sufferers to access appropriate support and medication, and therefore improve quality of life.

**Conclusion:**

These data can yield important insights into a substantial global healthcare issue and form the basis for improved patient awareness, professional education, clinical study design and drug development.

## Background

Headache disorders are among the main causes of disability worldwide; however, the majority of sufferers are never professionally diagnosed and instead, turn to over-the-counter (OTC) medications to self-manage symptoms [1]. While many other diseases decrease with socioeconomic development, worldwide analysis suggests that migraine and tension-type headache (TTH) are on the rise [1, 2]. Though predominantly experienced by those aged 15–49 years, headache incidence in school aged children is increasing, indicating that headache disorders are being reported earlier in life than they were before [1, 3]. There is accumulating evidence in recent literature that modern lifestyle in industrial countries may have an effect on headache incidence, prevalence and impact. In this short communication, we briefly analyse the phenomenon of ‘21st century headache’.

## Headache triggers associated with 21st century lifestyle

While general lifestyle factors, such as poor diet, stress and posture, are known causal factors, other aspects of modern life also influence headache disorders [1]. The recently increased use of digital technology is associated with increased risk of obesity, fatigue and headache; thus, headache incidence has been linked to prolonged (> 8 h/day) computer use in IT professionals in China and to excessive (> 4 h/day) video game use in adolescents in Brazil [4, 5]. Additionally, increased smartphone usage has been linked to headache, sleep disturbance, cognitive impairment and fatigue, with call frequency significantly correlated with headache risk [6].

More recently, the coronavirus disease 2019 (COVID-19) pandemic has been associated with both increased and decreased headache frequency [7]. In addition to being reported as a symptom of infection, headache frequency and severity increased in uninfected individuals due to psychological stress, social isolation, sleep disruption and poor dietary habits [8, 9]. New laws and policies introduced to mitigate the spread of COVID-19 have inevitably increased our dependence on digital technology, with working from home, online education and socialisation leading to increased average screen time [10]. The pandemic may therefore have unexpected consequences in terms of headache frequency, and future studies will determine the full extent of these consequences.

## Headache impact on cognitive and daily functionality

Both migraine and TTH negatively impact aspects of ‘mental clarity’, such as concentration, attention, reading, processing speed and memory [11, 12]. The cognitive impact of migraine has been well characterised, with multiple studies showing that migraine sufferers experience greater memory deficits during an attack compared with other headache types; however, the evidence for the impact of TTH on cognitive functioning is limited [11, 13]. One study has shown that TTH affects psychomotor performance and is associated with reduced quality of life [11].

Headache disorders contribute to distraction and poor concentration that define presenteeism at work; recent studies in Europe showed that only 50% of headache sufferers with presenteeism completed their normal working day [14, 15]. Absenteesim is also a problem: approximately 22% of migraine sufferers and 10% of TTH sufferers take several days per year off work due to headache [16]. Unsurprisingly, in line with the rising incidence of headache, the headache-associated years of life with disability have been increasing worldwide since 1990 [1]. The cognitive impact and loss of productivity may also be linked to anxiety, avoidance behaviour, reduced social interactions and lifestyle compromise reported by 16% of migraine sufferers and 20% of TTH sufferers [17, 18].

## Changing the paradigm: non-doctor headache

Although effective treatments exist, studies have shown that approximately 60% of migraine suffers and 80% of TTH sufferers never seek medical advice [16, 19, 20]. There is global variation in access to adequate healthcare, availability of medication, education and specific treatment guidelines, with headaches generally considered to have low priority in public health systems [1]. Alongside headache severity and frequency, the rate of healthcare utilisation is influenced by demographic and socioeconomic factors, such as age, occupation and status [21]. Often, sufferers attribute headache to muscular tension or everyday life situations, such as stress, relationships and hormonal fluctuations, and therefore believe that medical care is unnecessary [20]. This population of headache sufferers, that could be referred to as the ‘non-doctor treated headache’ (NDH) population, relies on OTC medication for symptom relief [22]. Consequently, there are limited data on the management of headache in the NDH population with respect to headache type, reduction in quality of life, triggers, OTC medication and non-pharmacological management techniques [23].

While randomised controlled trials (RCTs) adequately assess professionally diagnosed and managed headache, the NDH population should be captured by real world evidence studies, a number of which have been successfully undertaken and delivered important insights in headache-related behaviour and experiences [24]. These studies highlighted the need to understand better the NDH population and to develop strategies to engage and educate headache sufferers. Here, the digital technology can serve as a double-edged sword: while possibly attributing to rising incidence and prevalence of headache, it provides an enormous pool of real world data. An increasing number of smartphone applications capture and record headache frequency, intensity, triggers, duration and medication choice [25]. The accumulation of such data via freely available smartphone apps could predict headache days and allow tracking of headache-related impact on activities, productivity and quality of life [25]. Such technology could also be used to deliver non-pharmacological therapies, such as relaxation techniques and cognitive behavioural therapy [26]. Researchers studying cardiovascular diseases and diabetes have embraced the possibility to collect real world data directly from patients without time-consuming clinical visits and are becoming increasingly aware of the possibilities that smartphone applications may offer for self-management of chronic conditions [27, 28]. Of particular importance in this regard would be collection of information on relevant comorbidities, particularly pain syndromes, which may contribute to overall disability [29, 30]. Contemporary technology allows the integration of data in real time using state of the art analysis techniques (such as artificial intelligence) in a novel approach termed infodemiology. The time is ripe to use 21st century technology to map the new territory of 21st century headache.

## Conclusions

**Fig. 1:**
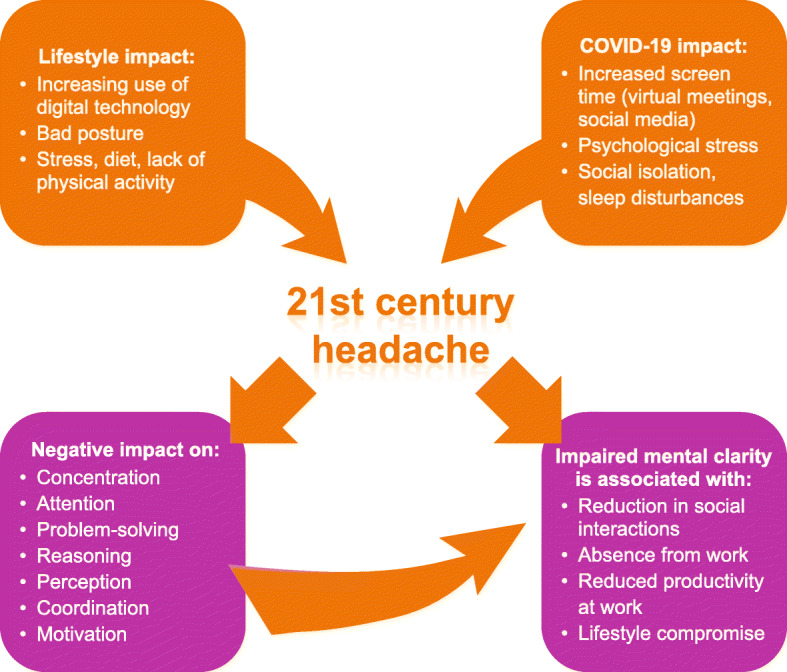
Triggers, suspected impact and burden of 21st century headache

Headache prevalence is reportedly increasing among all age groups, and the cognitive burden on individuals’ mental clarity comes at a cost to society as a whole (Fig. 1). Because causal factors seem to evolve with socioeconomic development, it is important to identify the true burden and triggers related to ‘21st century headache’ in real world settings [1, 3]. By exploiting widely available digital technology, such as smartphone apps, population-based real world data can be collected in real time to enhance our knowledge of triggers, impact and self-medication practices. Clinical experts and modern analysis techniques, such as artificial intelligence and machine learning, should be engaged in the analysis of these data to help identify NDH-relevant and specific outcome measures that should be further validated in RCTs assessing the impact of 21st century headache on cognitive abilities, functionality and society. Ultimately, these studies should inform medical training and treatment guidelines for the NDH population. Providing new guidelines to trained pharmacists and establishing an educational programme for the general population will empower headache sufferers to manage better their condition and decrease the burden of 21st century headache.

## Data Availability

Not applicable.

## Abbreviations

OTC: Over-the-counter
NDH: Non-doctor-treated headache
RCT: Randomised controlled trial
TTH: Tension-type headache
